# Foreign Hospitals

**Published:** 1895-02-09

**Authors:** 


### FOREIGN HOSPITALS.

### HOSPITALS IN CHILE.

Hospital matters in the Republic of Chile undoubtedly are sadly in need of reform. There exist throughout the country some seventy public hospitals, many of them having incomes of their own, and most, if not all, receiving a certain amount of Government support; but it may be doubted if any of these institutions are at all up to a modern standard of efficiency. Besides these hospitals there are a large number of smaller institutions, dispensaries, &c., also more or less State-aided. Santiago possesses the only lunatic asylum in Chile, and also the only maternity hospital.

A few words of description of one or two of the larger hospitals may be interesting. There are four general hospitals in Santiago, the Chilian capital. The oldest of these is the San Juan de Dios Hospital. This institution was established so long ago as 1556, by Pedro de Valdivia, the conqueror of Chile; it was destroyed by an earthquake in 1647, and was rebuilt in 1702. It is a two-storey erection, surrounding a square court, the wards containing each from ten to thirty-six beds. The floors of the wards are of pine wood, and the walls whitewashed. The ventilation is defective, and in common with most Chilian hospitals, there are no "service rooms," neither are there any baths in connection with the wards. Most hospitals, however, boast of a general bath house. This institution is intended for males only; a large number of the cases admitted being casualties, owing to its central position. Dr. Luis Asta-Burnaga quotes the following statistics as giving "an idea of the capacity and mortality of the institution":—

| | 1890. | 1891. |
|---|---|---|
| Discharged | 3,314 | 5,604 |
| Died | 901 | 977 |
| In hospital | 256 | 287 |
| Total treated | 4,471 | 6,867 |
| Mortality | 20·3 per cent. | 15·6 per cent. |

The San Francisco de Borja Hospital for Women was built in 1772, but there have been alterations of late years. There is a maternity service attached to it.

A newer institution is the hospital of San Vincente de Paul, built on the one-storey pavilion system, but the buildings are too crowded together. There are 20 pavilions, each containing from 26 to 36 beds. Here again ventilation is bad, and the windows deficient in size. A recent addition of six new brick wards brings up the total of beds to nearly 600. Pay patients are received at this hospital, which has the best reputation of any in Chile; it is also a clinical school and a military hospital. The death-rate is much lower here than at either of the two previously mentioned hospitals.

A new hospital, hardly yet completed, is the Salvador, of which two wards only have so far been opened, intended principally for incurable cases, such as cancer and tuberculosis. The mortality here for the year 1891 reached a total of 30·3 per cent.

Valparaiso contains only one State general hospital, the San Juan de Dios. The site is a very limited one for the size of the buildings, which are a mixture of new and old clustered round a large chapel. The number of beds is about 600. Valparaiso, as a city, is somewhat more advanced in modern views, and two well-built wards have recently been added through the enterprise of Mr. Enrique Lyon, one of the administrators, the sanitary arrangements of which are good. The windows are ample and ventilation sufficient. Some of the wards at this hospital have not been painted or emptied for six years, and it is not therefore surprising to find that erysipelas is endemic therein. Statistics show a record of 6,711 patients treated in 1891, and a mortality of 22·79 per cent.

As an example of the way things are done in Chile, a new hospital on the pavilion plan was some little time ago erected at Valparaiso through the generosity of a charitable lady, but owing to the want of knowledge displayed in its construction, when completed at a heavy cost, it proved entirely useless of the purpose of nursing the sick, and has consequently been turned into an orphan asylum.

The religious element interferes to no small extent with improvements in Chilian hospitals. Altars block up spaces in the wards which should be used for ventilation, and expensive and elaborate chapels crowd up the buildings where light and air are sorely needed to aid the recovery of the sick. Reform in these matters is naturally difficult to accomplish, seeing that the "Boards of Charity" who supervise the administration are mainly composed of members who have little or no special knowledge, and it is indeed a saying in Chile that no other qualification is needed for members of the Charity Board than to be "old, rich, and devout." One cannot help thinking that a little science thrown in might act usefully by way of leaven.

The nursing in all the hospitals throughout Chile is in the hands of Roman Catholic sisters.

